# Factors for the implementation of the circular economy in Big Data environments in service companies in post pandemic times of COVID-19: The case of Colombia

**DOI:** 10.3389/fdata.2023.1156780

**Published:** 2023-04-03

**Authors:** Carlos Alberto Almanza Junco, Marial del Pilar Pulido Ramirez, Mercedes Gaitán Angulo, Melva Inés Gómez-Caicedo, Álvaro Luis Mercado Suárez

**Affiliations:** ^1^Facultad Ciencias Económicas Campus, Universidad Militar Nueva Granada, Bogotá, Colombia; ^2^Facultad de Ciencias Administrativas, Económicas y Financieras, Fundación Universitaria del Área Andina, Bogotá, Colombia; ^3^Escuela de Negocios, Universidad Carlemany, Sant Julià de Lòria, Andorra; ^4^Facultad de Ciencias Económicas, Administrativas y Contables, Fundación Universitaria Los Libertadores, Bogotá, Colombia

**Keywords:** Big Data (BD), circular economy (CE), adoption intentions, TOE model, service SMEs, post pandemic

## Abstract

In emerging economies, Big Data (BD) analytics has become increasingly popular, particularly regarding the opportunities and expected benefits. Such analyzes have identified that the production and consumption of goods and services, while unavoidable, have proven to be unsustainable and inefficient. For this reason, the concept of the circular economy (CE) has emerged strongly as a sustainable approach that contributes to the eco-efficient use of resources. However, to develop a circular economy in DB environments, it is necessary to understand what factors influence the intention to accept its implementation. The main objective of this research was to assess the influence of attitudes, subjective norms, and perceived behavioral norms on the intention to adopt CE in BD-mediated environments. The methodology is quantitative, cross-sectional with a descriptive correlational approach, based on the theory of planned behavior and a Partial Least Squares Structural Equation Model (PLS-SEM). A total of 413 Colombian service SMEs participated in the study. The results show that managers' attitudes, subjective norms, and perceived norms of behavior positively influence the intentions of organizations to implement CB best practices. Furthermore, most organizations have positive intentions toward CE and that these intentions positively influence the adoption of DB; however, the lack of government support and cultural barriers are perceived as the main limitation for its adoption. The research leads to the conclusion that BD helps business and government develop strategies to move toward CE, and that there is a clear positive will and intent toward a more restorative and sustainable corporate strategy.

## 1. Introduction

In recent decades, living standards have risen because of industrialization. However, this has resulted in a serious impact on the environment due to the increase in the carbon footprint and the development of unsustainable production and consumption habits.

The growing carbon footprint and demand for natural resources thus becomes a challenge for economies, making it imperative that organizations work tirelessly to identify and mitigate the detrimental environmental effects of overconsumption (Dabbous and Tarhini, 2019; Shayganmehr et al., 2021; Abbate et al., 2023a). One way to identify and assess the environmental effects of organizations is the inclusion in organizational practices of increasingly Industry 4.0 (I4.0) and Circular Economy (CE) concepts (Luthra and Mangla, 2018; Frank et al., 2019).

The internet of things, Big Data (BD) technology, smart industries, additive manufacturing and robotic systems are the main technologies created by I4.0. It is generally accepted that these technologies have the capacity to solve problems of unsustainable production and consumption. According to Frank et al. (2019), the overall sustainability benefits of Industry 4.0 are expected to include increased productivity, flexibility, and resource efficiency. Big Data is expected to improve predictive maintenance and rapid reconfiguration of production systems and reduce waste, energy consumption and overproduction (Kiel et al., 2017). However, studies on digital transition and technology adoption identify this activity as a great gap in both management and operation (Abbate et al., 2022).

On the other hand, the ethical duty to produce based on sustainable, restorative and regenerative business processes and activities is gaining ground (Kirkire and Rane, 2017; Lin, 2018; Jabbour et al., 2020), with the promotion of production and sustainable consumption (Tseng et al., 2013, 2016) based particularly on the strongly emerging 3Rs concept of recycle, reuse, reduce (Shi et al., 2017; Tseng and Bui, 2017; Tseng et al., 2018a).

According to the Mundial (2014), the circular economy (CE) takes a restorative and regenerative approach to economic operations, increasing the overall sustainable performance of businesses by conserving and enhancing natural resources and recycling materials and by-products. Compared to current business models, CE places a greater emphasis on minimizing resource use and waste, and rather than taking a reactive stance to protect the environment from harmful business impacts, CE takes a proactive approach to developing systems self-sufficient that encourage reuse and recycling paradigms (Genovese et al., 2017; Jabbour et al., 2020).

In addition to the above, metrics, indicators and tools have also been developed to help in making sustainability decisions, and although they can be applied to various sectors and sizes of companies, the volume, variety, speed, and veracity of the data can in sometimes make decision making difficult. Efforts apply these concepts, limiting opportunities for sustainability improvement. In the context of this limitation, BD is presented itself as a possible solution to promote the deployment of a new generation of CE initiatives, especially in relation to the reduction of the intensity of use of raw materials, the reuse of products and the increase of the efficiency.

Today, to increase efficiency and growth, companies are looking for data-driven options (Tabesh et al., 2019), so BD has the potential to influence all business sectors and operations with lasting and long-lasting effects (Amankwah-Amoah, 2016). In this sense, BD can be used to understand organizational requirements and activities, simplifying, and developing sustainable business systems by facilitating informed decision making that can help implement sustainable business practices in accordance with the principles of the CB concept. Therefore, the combination of BD and CE has become crucial to facilitate profitable and sustainable production (Van Loon and Van Wassenhove, 2018; Dubey et al., 2019; Gupta et al., 2019) and to increase efficiency and compete globally (Garcia-Muiña et al., 2018; Rajput and Singh, 2019; Dantas et al., 2021).

Although recycling and BD are currently highly promoted, only about 9% of waste is recycled, and < 10% of companies make heavy use of BD (Jia et al., 2020), so increasing this figure is necessary not only for technical solutions or organizational planning on the subject, but also to understand the human behaviors that are generated in the action of recycling as well as the factors that influence the adoption of BD. In this regard, although the literature is extensive in the progress of these two topics, there are no known studies on the case of Colombia that evaluate the acceptance of the implementation of a circular economy in BD environments through multivariate analyzes that are addressed in this article.

The objective of this study is to evaluate the influence of attitudes, subjective norms, and norms of behavior on intentions toward CE in BD environments in a total of 413 service SMEs located in Colombia, due to the fact that currently, although it is an issue recurring in the specialized literature. No studies have been carried out on the subject in emerging Latin American economies, and since a large part of these economies generate raw materials and are responsible for the Amazon, no studies have been carried out on the subject in emerging Latin American economies, and given that a large part of these economies are responsible producers of raw materials, as in the case of Colombia, from the Amazon, any academic effort to minimize environmental impacts will be valued both by the businessmen and by humanity in general.

The organization of the article is as follows: Section 2 presents a review of the literature, including different concepts of BD and CE. Section 3 presents the theoretical framework and the development of the hypotheses. Section 4 links the methodology, with a description of the instrument, the sample and the data process. Section 5 presents the results. Section 6 provides conclusions with theoretical, practical, and social implications and recommendations and Section 7 provides limitations and future research directions.

## 2. Literature review

For humanity, but especially for companies, sustainability has gone from being a variable or slogan of good intentions to an urgent need when it comes to growing and surviving. The influence of this variable in the definition of business plans and policies has allowed the incorporation of sustainable practices and initiatives not only at the procedural level in employees (Abid et al., 2020; Naqvi, 2020), but also the entry of increasingly frequent CE efforts in companies, especially large ones (Ajwani-Ramchandani et al., 2021; Morea et al., 2021), which, by the way, generate significant environmental pollution (Choi et al., 2020; Pelletier et al., 2020; Rai et al., 2020; Ribeiro-Brasil et al., 2020).

Solving this pollution problem is not easy, fast, or cheap, since it requires efficient management of waste, usually plastics, that reduces pollution and at the same time avoids the loss of resources, so the concept of CE can be very useful in this regard. In this sense, the EC is aligned with SDGs 11 (sustainable cities and communities) and 12 (responsible production and consumption) in the sense that it assumes that waste can be maintained without releasing it into the natural environment, which has aroused interest from a wide variety of applications, including plastic waste management (Fletcher et al., 2021), hospitals (Chauhan et al., 2021), ports (Roberts et al., 2021), automobiles (Kamble et al., 2021), the Internet (Yadav et al., 2020), and the use of plastic as a waste management material (Yadav et al., 2020; Kamble et al., 2021), Internet of Things (Cavalieri et al., 2021), textiles (Jia et al., 2020) and supply chains (Dev et al., 2020).

Sadeghi Ahangar et al. (2021) indicate that waste management is considered an important issue on government agendas, especially in emerging economies, which includes the collection, separation, transfer, disposal, and recycling of waste. In addition, the amount of solid waste has increased in recent years, which can be attributed to several reasons, including increased production in factories, increased world population, with the mitigation that more than 50% of the world population lives in cities. Therefore, having a proper supply chain for disposal and recycling is preventing environmental pollution to a great extent.

By repurposing waste and offering sustainable solutions, CE transforms the traditional linear economic view from a take-make-use-dispose cycle to a take-make-use-recycle approach. According to Sahu et al. (2021), the EC bases its efforts on three guiding principles: the conservation of natural assets, the increase in the circularity of resources and the reduction of adverse effects on the system and the environment. Some common strategies in the application of the circular economy are repair and maintenance, reuse and redistribution, restoration and remanufacturing, recycling and reuse, so it is understandable that CE can eliminate the problems of scarcity of resources and help create value for the company (Bag et al., 2021).

### 2.1. From the linear to the circular economy

The idea of linear economy, which forms the basis of many contemporary economic models, emphasizes product acquisition, use, and disposal, ignoring reinvestment in production and/or consumption cycles (Murray et al., 2017; Corvellec et al., 2022). This model has also led consumers to develop a linear consumption behavior (Lieder and Rashid, 2016; Garcés-Ayerbe et al., 2019; Velenturf and Purnell, 2021), which makes it increasingly necessary to adopt sustainable strategies and respectful with the environment, to restore the growing ecological imbalance and social wellbeing (Dantas et al., 2021).

Although the concept of a sustainable business approach is not particularly new, it has attracted a lot of attention lately, especially in terms of increasing productivity, reducing environmental degradation and increasing consumer awareness of the goods they buy (Hameed et al., 2019).

According to Gupta et al. (2019), linear economic models treat businesses as open-loop systems in which natural resources are continuously obtained and consumed, without concern for their final disposal, which generates large amounts of waste and contamination (Gupta et al., 2019). In contrast, CE operates on the fundamental principle that any business cycle should synergistically integrate production activities, so that waste or by-products from one level can be used as raw material for the next, giving its restorative and restorative connotation (Gupta et al., 2019; Abbate et al., 2023b).

While the CE model has many benefits for sustainability and resource use, it also presents a number of challenges for organizations. By adopting a CE model, current organizational models and capabilities may become outdated and ineffective, which could lead to radical and systemic improvements (Bocken et al., 2019; Pieroni et al., 2019). CE models require simultaneous coordination of all relevant systems and networks to ensure that all stakeholders benefit (Van Langen et al., 2021). Decision-making is then crucial to maximize performance and reduce leakage and system externalities. And since they need to be backed by timely and accurate information, data collection and analysis become a crucial challenge for CE systems, so DB will play a relevant role here.

### 2.2. Circular economy (CE)

The literature on CE is extensive and growing, ranging from reports on green awareness (Liu and Bai, 2014) to the detailed study of the behavior of companies regarding the development of enablers (Gusmerotti et al., 2019) or inhibitors of EC (García-Quevedo et al., 2020). Progress has also been made in the study of business models (Scarpellini et al., 2020), the potential of technology (Jakhar et al., 2018) and the role of digital technologies in the implementation of CE (Gaustad et al., 2018). In this regard, Giudice et al. (2020), provide an exhaustive review of the progress of the relationship between CE and Industry 4.0.

A relevant aspect of the studies is that they have focused their efforts on finding out not only the most widely used strategies to implement CE (Ranta et al., 2021) but have also sought to identify which elements make it possible to move from a linear to a circular economy (Garcés-Ayerbe et al., 2019).

For example, Lerdlattaporn et al. (2021) shows how the starch industry can have sustainable production by converting cassava pulp and wastewater into biogas. Norouzi et al. (2021) also shows how the construction sector has benefited from the development and use of alternative construction materials and the design of smart cities.

Other initiatives have identified how environmental commitment and green economic incentives are predictors and facilitators of CE (Singh et al., 2018); while the lack of capital, government support, information, technical and technological knowledge and the company's environmental culture have become the main barriers faced by SMEs when implementing CE business models (Rizos et al., 2016). In this sense, tools such as Match (making the transition to a circular economy) have helped organizations through self-assessments to identify the primary conditions to make the transition from linear to circular (Pigosso and McAloone, 2021).

The literature also shows that, in relation to clients, subjective norms, willingness to sacrifice for the good of the environment, perceived economic benefits, and expected positive emotions can influence citizens' willingness to participate in CE (Hao et al., 2020). Similarly, when reviewing consumer groups perceptions of remanufactured products, studies show that when consumers are environmentally friendly or perceive recycled products as environmentally friendly, they tend to find recycled products more attractive (Abbey et al., 2015). According to Gaur et al. (2015), the level of environmental awareness, personal values, post-consumption perception, the nature of the purchase and socio-cultural norms are important drivers of consumer willingness to buy recycled products. Similarly, Gaur et al. (2015) identifies that contextual factor such as price, promotion/advertising, service quality and brand image, although influential, are not determinants of the consumption of products that are part of the circular economies.

### 2.3. Big Data (DB)

The rapid advancement of contemporary technology, such as the Internet and cloud computing, has unleashed new resources in the form of data, both from individual and collective processes. These data that are distinguished by volume, diversity in terms of geographic scope or activity categories, and by the speed with which they are generated are known as Big Data (Jin et al., 2015). But BD is more than a bunch of data. BD is characterized by comprehensive data management that is combined from a variety of sources and is available in real time. This poses a new challenge: how to manage the potential informational value of data to generate competitive advantage. This value can be achieved with the help of BD, where all methods and technologies are used to analyze large amounts of heterogeneous business data and provide useful information for decision making (Ferraris et al., 2018; Merendino et al., 2018). Therefore, decision-making in today's complex economic activities, including those of CI, will undoubtedly be favored by this type of comprehensive analysis.

#### 2.3.1. Use and applications of Big Data (DB)

Database analytics is a novel approach that, by identifying patterns in topic-oriented data sets at a specific time, helps make sound decisions, increase productivity, and create knowledge (Gonzales et al., 2015; Jin et al., 2015; Staegemann et al., 2021).

Typically, BD reflects interactions between employees, consumers, suppliers, and distributors of products (goods or services), which often provide descriptive and predictive results (Shirdastian et al., 2019; Baig et al., 2021), which drastically transform the competitiveness of firms, depending on the acquisition of timely information to achieve higher levels of performance (Gonzales et al., 2015; Volk et al., 2020).

The use of BD as a solid foundation for performance improvement has been established in Coleman et al. (2016); Akter et al. (2017); Mikalef et al. (2019); Almaiah and Nasereddin (2020), and Dubey et al. (2020) and most of these studies indicated a favorable association between DB success and business performance. However, due to the large size of the database, obtaining important information and insights remains a challenge (Volk et al., 2020).

BD has been used by large corporations for a number of purposes, including, but not limited to, identifying new opportunities for improvement and forecasting future market trends, and while many companies consider the adoption of BD critical and believe it has great potential (Verhoef et al., 2016; Staegemann et al., 2021), the literature reveals that its adoption has not only been quite modest (Nam et al., 2019) but that many companies have not achieved integrated use beyond the process early adoption (Choi et al., 2022) or adequate results (Almaiah and Nasereddin, 2020; Al-Sai et al., 2020).

The literature review also reveals that research on the adoption of BD in small and medium-sized enterprises (SMEs) is incipient (Al-Sai et al., 2020; Dubey et al., 2020; Munawar et al., 2020) and that although they contribute significantly to the national economy, they lag far behind in DB implementation due to limited resources and lack of awareness of the main barriers to implementation (Nam et al., 2015; Coleman et al., 2016; O'Connor and Kelly, 2017; Ghasemaghaei, 2019).

Therefore, the purpose of this article is to explore the factors influencing DB adoption in the SME sector using a technology, organization, and environment (TOE) paradigm. Since it flexibly describes the degree of technology adoption in various companies, the TOE model is appropriate for application in this situation (Chandra and Kumar, 2018; Maroufkhani et al., 2020).

The impact of TOE factors on technology adoption and innovation has been the subject of numerous studies (Lutfi et al., 2016; Althunibat et al., 2021), but the results do not apply to DB adoption by SMEs as the TOE model factors are influenced and determined by the type of technology (Gangwar et al., 2014; Alharbi et al., 2016; Sun et al., 2020), firm size (Brozovic, 2018), resource availability (Maroufkhani et al., 2020) and study context (Alharbi et al., 2016; Al-Sai et al., 2020). In this regard, the following study aims to contribute to the body of knowledge on TOE factors influencing DB adoption in SMEs with the hope that the results will help SME managers to understand these adoption factors for decision making.

Therefore, there is a clear need to investigate and understand the drivers of DB adoption by SMEs, since according to Al-Sai et al. (2020) it is a novel topic with few empirical results, which is also focuses on what happens in developed countries (Frizzo-Barker et al., 2016). However, it is important to note that several authors have highlighted the need to investigate the adoption of DB (Agrawal et al., 2015; Sun et al., 2018; Wahab et al., 2021), especially in developing countries, to expand the existing literature (Al-Sai et al., 2020).

#### 2.3.2. Factors associated with the adaption of Big Data

The expansion and development of social media, ecommerce, cutting-edge mobile technologies, search engines and new digital technologies have produced an increasing amount of business data, paving the way for businesses to collect and capture this data to examine and generate patterns of information relevant to their performance (Merendino et al., 2018; Ghasemaghaei, 2019).

The use of BD in SMEs has been the subject of several research studies. For example, Ajimoko (2018) addresses the key requirements for DB implementation based on three models: innovation diffusion theory, technology acceptance model, and technology-organization-environment (TOE) model. The results classify the crucial adoption factors into internal and external groups. External criteria include environmental and supplier-related aspects, which have less influence on DB implementation. Internal criteria include technical and organizational elements, which are crucial in DB adoption.

Mangla et al. (2020) used the SEM approach to show how the use of DB would improve project performance and sustainability. Nine factors were analyzed including project operational capabilities, social responsibility, environmental technologies, green contracting and a project knowledge management approach, sustainability, senior management, project success, exploratory learning, collaboration, and the complexity of the project.

Nasrollahi et al. (2021) conducted similar research on SMEs in Iran and found that DB adoption positively affects SME performance, in addition, they identified that social performance, economic performance and operational performance have a substantial influence on the implementation of DB.

In the same way, Maroufkhani et al. (2020), analyzed the crucial factors for the adoption of DB, and the effect it has on the productivity of SMEs. Their findings showed that BD adoption in SMEs was significantly affected in particular by seven factors, including top management support, observability, testability, uncertainty and insecurity, complexity, external support and organizational preparation. They also found that using of DB significantly improves performance.

Park et al. (2015) also conducted research to determine the crucial factors of BD adoption. Based on the results, the adoption categories can be divided into three: environmental, organizational, and technological (TOE). Using this same TOE approach, Skafi et al. (2020) examined Lebanese companies and found that technical aspects such as security and complexity have a favorable impact on DB adoption and that organizational aspects, such as previous experience in IT and the support of senior management, had a positive impact on the DB adoption decision.

To help Malaysian companies improve their performance and overcome obstacles during lockdown, Loh and Teoh (2021) focused on how technical variables have affected DB adoption in SMEs. This study found that technological factors might motivate companies to successfully adopt and use the DB more than organizational factors.

### 2.4. Theory of planned behavior of TCP

The invention of more and more products has made modern life much easier. However, this massive growth of products has generated a maelstrom of waste that is now considered an *environmental* scourge (Plummer, 2018). Despite growing concern about the environmental issue, production continues to increase, and it is estimated that by 2023 >1,400 million new products will be generated worldwide each year, which affects the consumption of non-renewable resources such as oil, since these new products absorb between 8 and 10% of the total world extraction (Leal et al., 2021).

To overcome these unfounded fears, the CE concept is gaining momentum as a promising solution. However, while many countries encourage the implementation of CE practices, the fact is that only 9% of all waste was recycled worldwide between 1950 and 2015 (Geyer et al., 2017), so the potential for waste recycling has not yet been tapped. However, it is worth noting that this problem cannot be addressed only with technical solutions or visions, since it also involves acquired behaviors and psychological traits that often hinder the continuity and viability of technical solutions, which requires greater and better understanding of human behavior associated with recycling activity (Heidbreder et al., 2019). It is in the context of understanding these behaviors that Ajzen's (1985) theory of Planned Behavior becomes relevant.

The Theory of Planned Behavior (TCP) is considered one of the most powerful models for predicting behavior (Yuriev et al., 2020). The PCT proposes that attitude, subjective norms, and perceived behavioral control define intention regarding a specific behavior and this intention becomes the strongest predictor of actual behavior (Hill et al., 1997; Yuriev et al., 2020). This particularity has led CTP to be associated with sustainability management and pro-ambivalent behaviors (Daddi et al., 2019; Si et al., 2019).

Many studies have used MCT to understand human recycling intention and behavior (Nigbur et al., 2010; Ramayah et al., 2012; Botetzagias et al., 2015; Khan et al., 2019). Such studies have focused their efforts on understanding or predicting outcomes, particularly at the individual level in households (e.g., household heads and households) or academic institutions (e.g., adult learners) (Geiger et al., 2019) and have rarely targeted organizations (e.g., managers or employees) to understand managers' waste recycling intentions and behavior's and predict organizational level outcomes (Yuriev et al., 2020).

To close this knowledge gap, the research focuses specifically on organizations through their managers for two reasons. First, organizations generate a large proportion of waste, and while much of this waste is technically recyclable, it often cannot be properly collected or recycled because organizations are not connected to the proper systems (Antonopoulos et al., 2021). Second, managers are key to the adoption of circular recommendations, as their individual perceptions, attitudes, and values often influence the organization's strategic actions regarding CE, so managers intentions are the intentions of organizations (Daddi et al., 2019; Gusmerotti et al., 2019).

## 3. Theoretical framework and hypothesis

### 3.1. Theory of planned behavior regarding the intention of the circular economy

According to CTP, intention toward a specific behavior is determined by attitude, subjective norms and perceived behavioral control (Ajzen, 1985). CTP understands by attitude the degree of favorability or unfavourability of a person toward a certain behavior. This attitude can then strengthen or weaken the intention of that individual to perform a certain behavior (Khan et al., 2020a). For this paper, attitudes refer to the extent to which managers value recycling, either positively or negatively. Those who have positive attitudes toward recycling are more likely to implement recycling best practices in their organizations (Khan et al., 2020a).

The relationship between the subjective norm and behavioral intention is widely recognized in the literature (Chen and Tung, 2010; Khan et al., 2020b).

For this work, the subjective norm is reviewed considering the social norms for recycling, which by the way can vary according to the cultural, social, or economic context. For example, what in one sector may be mandatory in others is only a recommendation, so the intention of a manager's behavior toward CE can be influenced by whether companies, communities or neighboring countries support these practices or not (Thoradeniya et al., 2015). In this sense a manager's intention can only be affected by whether others in his organization are in favor of recycling. According to this article, subjective norms are understood as the degree to which managers are influenced by perceived recycling norms in their organizational environment, this presupposes that managers who perceive positive social norms about recycling are more likely to implement recycling best practices in their organizations.

Finally, Ajzen (1991) understands perceived behavioral control as the perceived ease or difficulty of performing the behavior, and as a subjective norm the relationship between perceived behavioral control and behavioral intention is documented (Han and Yoon, 2015; Khan et al., 2019). For this paper perceived behavioral control is understood as the perceived power of managers to implement recycling, this implies who have strong perceived behavioral control are more likely to implement recycling best practices in their organizations.

Although previous studies have used CCT to address human intention related to environmental problems and recycling behavior (Oztekin et al., 2017; Aboelmaged, 2020; Mak et al., 2020; Wan et al., 2021), little has been addressed with respect to considering a sample of organizational managers to predict organizational level outcomes regarding behavior toward a CE. In this novel approach, efforts will focus on understanding the role of companies, since they generate a representative proportion of waste that is often not properly managed and because most companies are not involved in CE. Another reason to approach CTP is that the CE involves individual attitudes, perceptions, and values, so the use of this latent variable can identify the intentions and behaviors of the so-called decision-makers in companies.

#### 3.1.1. Attitude

The degree to which a person has a favorable or unfavorable opinion or evaluation of an action or behavior is defined according to Ajzen (1991) as an attitude. For Arli et al. (2020) there is a strong relationship between attitude and behavioral intention, and since from the MCT perspective, attitude is the best predictor of behavior, the present study investigates the degree to which decision-makers value, positively or negatively, the CE and its components:

Hypothesis 1 (H1):

Managers' attitudes positively influence the intentions of organizations to implement CE practices.

#### 3.1.2. Subjective standard

The subjective norm according to Ajzen (1991), is understood as the perceived social pressure on an individual to carry out or not perform a certain behavior. The subjective norm is determined by two components: on the one hand, the perception that other people generally important to the subject expect and approve of such behavior; on the other hand, the subject's own motivation to conform to the expectations of these people (Ajzen, 1991). According to Chen and Tung (2010) there is a relationship between subjective norm and the behavioral intention, so it is expected that:

Hypothesis 2 (H2):

The subjective norms perceived by managers positively influence the intentions of organizations to implement CE practices.

#### 3.1.3. Perceived behavioral control

Perceived control is defined as the perceived ease or difficulty of performing the behavior (Ajzen, 1991). Some research supports the relationship between perceived behavioral intention (Tonglet et al., 2004; Parajuly et al., 2020). The current study considers perceived behavioral control as the perceived power of decision-makers to recycle.

Hypothesis 3 (H3):

The manager's perceived behavioral control positively influences the intentions of organizations to implement CE practices.

#### 3.1.4. The intention of the circular economy

The intention to perform a certain behavior is a prior state of the behavior as such (Ajzen, 1991). For Pisitsankkhakarn and Vassanadumrongdee (2020) and Coderoni and Perito (2020) it is during this previous state that the individual, based on interest, creates reasons to carry out a certain behavior. Although some factors influence the intention to act, ultimately it is the individual who, based on the realization of his expectations of him, decides whether to act in one way or another. This suggests that intent is a necessary and antecedent factor for any desired behavior, so if intent can be accurately measured, companies can largely anticipate and predict actual expected behavior (Ajzen, 1991; Coderoni and Perito, 2020). The present study considers the intention of CE behavior as a trigger for the development of CE capabilities. Consequently, it is proposed:

Hypothesis 4 (H4):

The intention of CE positively influences the capacities of organizations to implement CE practices.

Although the traditional components of the TCP model (attitude, subjective norms, and perceived behavioral control) have been successful in predicting human intentions and behavior's, many researchers argue that additional variables should be incorporated into the TCP model to improve its explanatory power (Chen and Tung, 2010; Botetzagias et al., 2015; Singh et al., 2018; Geiger et al., 2019; Khan et al., 2020b).

Ajzen (1991) points out that the CTP is open to the inclusion of additional predictors considering the context and purpose of their studies, particularly in the intention-behavior relationship. Therefore, the TCP model is modified by incorporating elements of the TOE model (technological and organizational environmental factors) as direct factors that can influence the circular economy behaviors and capabilities of organizations. To incorporate additional constructs into the TCP model, the guidelines of Whetten et al. (2009) in relation to contexts and levels.

#### 3.1.5. Build circular economy capabilities driven by Big Data analytics

A company can benefit from the connection between CE (recycle, reduce, reuse) and Big Data (reliability, variety, speed, and volume) (Jabbour et al., 2020). For example, the choice of raw materials may be affected by the accuracy (truthfulness) of the DB in assessing the environmental impact of those inputs. In addition, the analytical capacity (variety) and the responsiveness in terms of time (velocity) of the information generated in any process (volume) will be crucial to creating effective CE plans. This means that in terms of e.g., tracking customer preferences and consumption patterns, the speed, veracity and volume of information could at any time change the CE capabilities in an organization (Dhamija and Bag, 2020; Jabbour et al., 2020).

According to Gupta et al. (2019), Big Data generally has a positive relationship with CE capabilities. This relationship has also been established by Stock and Seliger (2016); Theorin et al. (2017); Carvalho et al. (2018); Fisher et al. (2018); Lopes de Sousa et al. (2018); Stock et al. (2018) and Dubey et al. (2020). In addition, Tseng et al. (2018b) show that the circularity capabilities in terms of reduction, recycling and reuse of products or raw materials are enhanced under the protection of the DB, as it manages to identify patterns and new uses, which undoubtedly contributes to improve organizational results. Finally, Rajput and Singh (2019) and Nascimento et al. (2018) identified that DB is not only supports CE practices but also facilitates CE capabilities. Therefore, it is proposed:

Hypothesis 5 (H5):

DB adoption has a positive relationship with CE capabilities.

### 3.2. TOE Model (technological-organizational-environmental)

Several studies have identified that the use of the TOE model could help to examine the level of IT adoption in SMEs (Oliveira et al., 2019; Lutfi et al., 2020). In essence, the TOE model explains how factors (internal and external) can affect the adoption of technology in companies (Yoon and George, 2013). In this sense, the model can help to easily identify the most representative drivers of the successful adoption of DB in SMEs. Although several TOE factors have been shown to influence technology adoption, this study focuses on technological factors (relative advantage, compatibility, and security), organizational factors (top management support and organizational readiness) and environmental factors (competitive pressure and government regulations).

#### 3.2.1. Technological context: The comparative advantage

According to the literature (Rogers, 2003; Kapoor et al., 2015) the TOE model helps to identify internal and external elements of the technology in terms of its adoption. One of these elements is associated with the possible generation of relative advantage, which could directly affect the intention to adopt these technologies or not (Lutfi et al., 2021; Wahab et al., 2021). Relative advantage is usually understood as the level at which the adoption of a technology, as well as the benefits of its use, are perceived as better than other existing types of technology used in companies (Lutfi, 2021). According to Lutfi (2021), SMEs tend to adopt technology when they are convinced that its advantages are greater than those of any other existing technology; therefore, this article proposes the following hypothesis to be tested:

Hypothesis 6 (H6):

BD's relative advantage positively influences its adoption.

#### 3.2.2. Technological context: Compatibility

Compatibility is understood according to Awa et al. (2017) as the degree of alignment between a new system and the current system of a company. In terms of technology adoption, compatibility reflects the alignment of the technology with the business processes and culture of the organization (Kapoor et al., 2015; Baig et al., 2021). Gangwar et al. (2014) and Awa et al. (2017) state that compatibility is not only one of the main drivers of technology adoption, but also the most important criteria for determining BD, so flexibility in procedures and policies could encourage not only the intention to adopt DB (Gangwar et al., 2014), but also DB implementation. Taking the above, this study proposes the following hypothesis:

Hypothesis 7 (H7):

Database compatibility has a positive relationship with adoption.

#### 3.2.3. Technological context: Security

Security is another technology factor that can affect technology adoption. Security, according to Alshamaila et al. (2013), refers to the risk associated with the adoption of a technology. In this sense, Asiaei and Rahim (2019) stated that the adoption of a technology when it is related to data requires guaranteeing security, since privacy and security concerns, which are inextricably linked to the nature of the data, are predictors of the adoption of DB (Lai et al., 2018; Asiaei and Rahim, 2019; Ghasemaghaei, 2020). This concern for security is even greater when outsourcing to expert third parties, since the risks that arise in outsourcing, linked to the use of tools and support to provide a DB solution, significantly exceed the ability of the company to control the risks processes and confidential information, significantly affecting BD adoption; (Priyadarshinee et al., 2017). This situation is more common in medium and small companies due, firstly, to the lack of capacity to develop and maintain a DB environment and, secondly, to the lack of knowledge and innovation in DB-related technologies (Lai et al., 2018). In this line, this work proposes the following hypothesis.

Hypothesis 8 (H8):

High level of insecurity DB has a negative relationship with adoption.

#### 3.2.4. Organizational context: Senior management

Two central elements influencing DB adoption are top management support and organizational readiness. According to Afshar and Brem (2017) to the extent that managers understand and accept new technological capabilities, they are more likely to facilitate the adoption of new technologies. In this regard Alshamaila et al. (2013); Lutfi et al. (2016); Afshar and Brem (2017), and Cruz-Jesus et al. (2019), and identified senior management support in adopting new technologies as needed. Considering the above, this study proposes the following hypothesis:

Hypothesis 9 (H9):

Top management support has a positive relationship with BD adoption.

#### 3.2.5. Organizational context: Organizational readiness

Organizational readiness has been recognized by Gangwar et al. (2014) and Wahab et al. (2021) as necessary for the adoption of BD. According to the latter, organizational readiness is the ability and orientation of the company to manage and invest in the adoption of new technologies. In this regard Lutfi et al. (2016) and Asiaei and Rahim (2019) have shown that organizational preparation in SMEs has a significant association with the adoption of new technologies, so it is understandable to say that, for the adoption of DB, the organizational preparation is a central study variable and as such, the following hypothesis is proposed:

Hypothesis 10 (H10):

Organizational readiness has a positive relationship with DB adoption.

#### 3.2.6. Environmental context: competitive pressure

Xu et al. (2017) propose that the environmental context, although it is made up of multiple elements external to the organization to which it is sensitive, two are the most representative: competitive pressures and government regulations. According to Baig et al. (2021) competitive pressure is understood as the effects of the external environment, in this case suppliers, customers and competitors on a company that force it to be more competitive, which often leads to the adoption of new technologies (Asiaei and Rahim, 2019; Lutfi et al., 2020; Baig et al., 2021).

Recent studies show that competition between companies has a significant effect on technology adoption (Chen et al., 2015; Lautenbach et al., 2017), especially in the use of DB that can give advantages in terms of decision making, so this study proposes:

Hypothesis 11 (H11):

Competitive pressure has a positive relationship with the adoption of BD.

#### 3.2.7. Environmental context: Government regulation

Government regulations, on the other hand, can enhance or inhibit the adaptation of new technologies (Tornatzky et al., 1990; Lutfi et al., 2016). In this sense, the probability of DB adoption by companies could increase when regulations, government policies, tax benefits and incentives or legislation motivate them to do so (Lai et al., 2018). Therefore, this study proposes that when there are government regulations in the form of aid and incentives, the adoption of BD is stimulated; hence:

Hypothesis 12 (H12):

Government support has a positive relationship with the adoption of BD.

### 3.3. Research model

**Figure 1:**
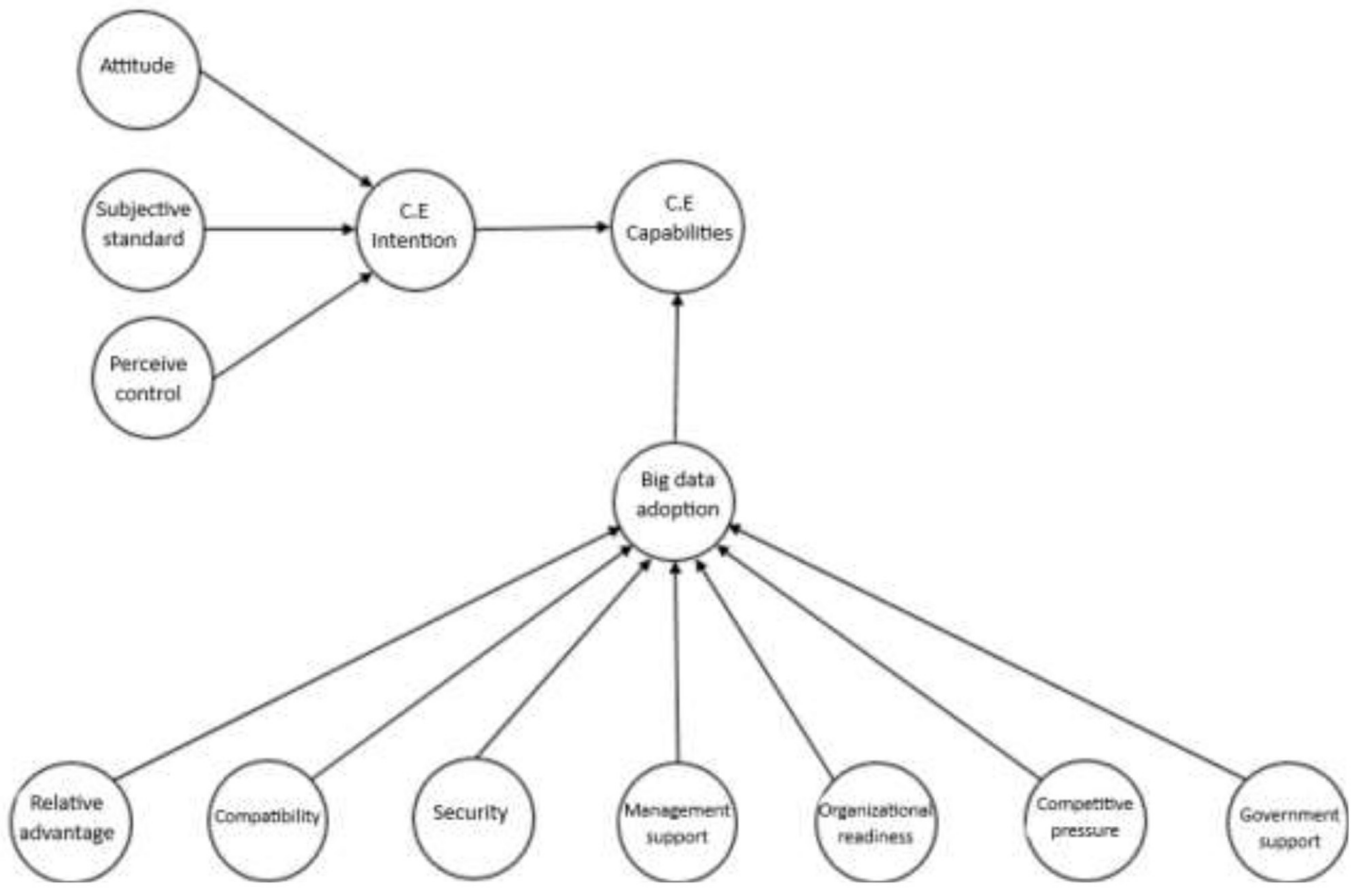
Proposed research model.

Figure 1 shows in detail the relationship between the study variables of the research model. The research model considers attitude, subjective norm, perceived control, intention regarding circular economy, and behavior regarding TOE factors with respect BD adoption.

## 4. Methodology

### 4.1. Data collection

The present study is a quantitative, cross-sectional, non-experimental, and descriptive study with an inference design, which aims to describe the influence of the factors that can explain the acceptance of the implementation of the circular economy in companies in Big Data environments.

For its development, a questionnaire was elaborated following the recommendations of Churchill (1979), where in the first place, relevant previous studies were identified and selected, followed by the identification of indicators related to the constructs, which implies the compilation and adaptation of indicators considering the context and purpose of the study. The third step was the writing of a test questionnaire, which was reviewed by five experts and adjusted according to their recommendations. Finally, the application and determination of psychometric properties was carried out to confirm the suitability and validity of the instrument (Hair et al., 2017).

#### 4.1.1. Sampling

The inclusion criteria were companies from the service sector in Colombia. The sample is made up of 413 Colombian service SMEs. A total of 637 surveys were sent with a response rate of 71% (484), of which 71 were rejected or being incomplete. The average number of years the company was created was 8.6 years (SD = 11.23) and 42.3 workers (SD = 17.53) (Table 1).

**Table 1:** Description of the sample.

**Characteristics of participating subjects**	
	%
Sex	
Man	63,27%
Woman	36,73%
	
Schooling	
Bachelor	11,25%
Technician	18,90%
Technologist	36,94%
University	25,27%
Postgraduate	7,64%
	
Age	
From 18 to 25	13,80%
From 26 to 30	28,45%
	
From 45 to 60	12,74%
More than 60	2,76%
	
Type of contract	
Fixed term	58,60%
Indefinite	19,96%
Outsourcing	21,44%
	
Seniority in the company	
Under 1 year	19,96%
From 1 and 3 years old	28,24%
From 3 to 5 years old	19,75%
From 5 to 10 years old	20,17%
More than 10 years	11,89%

### 4.2. Instruments and data collection

The questionnaire includes 48 questions based on the instrument used by Khan et al. (2019) circular economy and Lutfi (2021) big data adoption. The questionnaire includes questions based on the instrument used by Khan et al. (2019) circular economy and Lutfi (2021) big data adoption. The original articles were translated and adapted linguistically. The variables were measured using a 5-point Likert-type scale (from 1 = completely disagree to 5 = agree). The final version of the questionnaire was uploaded to Google Forms and distributed to company managers by email between May 16 and September 30, 2022. Managers filled out the online form anonymously following the ethical recommendations of the investigation.

### 4.3. Data analysis

Structural equation modeling (SEM) has been used for data analysis, which is considered a particularly powerful statistical method in this field (Hair et al., 2012a). SEM can be carried out using two different methods: partial least squares SEM and covariance-based SEM (CB-SEM) (PLS-SEM). When the study is exploratory and focuses on predicting events rather than discovering how complicated models are related to events, PLS-SEM is recommended (Hair et al., 2011). Also, most researchers employ PLS-SEM in studies related to CD and TCP because it can demonstrate more statistical power than CB-SEM (Khan et al., 2020b), for these two reasons PLS-SEM was preferred over CB-SEM (Sarstedt et al., 2019).

It is important to note that the sample size used in the study adheres to the general rule of thumb suggested by Hair et al. (2012b): ten times the number of indicators of the construct with the highest number of indicators.

SmartPLS version 3.3.2 was used to evaluate the collected data. For the internal consistency of the subscales, Cronbach's alpha reliability coefficient was used. Construct and discriminant validity, and internal consistency were assessed using composite reliability (Ringle et al., 2015). The reliability of each indicator was assessed by measuring the indicator loadings. The mean variance extracted was used to analyze the fit and finally the Fornell-Larcker criterion was used to assess discriminant validity (Fornell and Larcker, 1981).

Chin (2010) suggests a two-step process for examining and interpreting PLS-SEM; first, to evaluate the measurement model with 300 iterations of the PLS algorithm, second, to estimate the structural model using bootstrapping with 5000 subsamples. This study followed these recommendations by performing PLS-SEM analysis and disseminating the results (Chin, 2010; Hair et al., 2017).

## 5. Results

### 5.1. Reliability and validity of the measurement model

To evaluate the measurement model, the reliability, internal consistency, convergent and discriminant validity of the indicator were examined (Hair et al., 2017). According to Hair et al. (2012a), the load of an indicator must be greater than 0.70 and the average variance extracted (AVE) of each construct must be greater than 0.50 (Hulland, 1999; Hair et al., 2012a), for this study both the indicator loads, and AVE of each construct complied with these recommendations (see Table 2).

**Table 2:** Reliability and validity of the measurement model.

**Construct**	**Code of the indicator**	**Indicators**	**Factorial loadings ^1^**	**Cronbach's alph*a^2^***	**C*r^3^***	**AV*E^4^***
Attitudes (Act)	Act1	Recycling is good	0.858	0.888	0.888	0.651
	Act2	Recycling is useful	0.868			
	Act3	Recycling is rewarding	0.941			
	Act4	Recycling makes sense	0.839			
	Act5	Recycling would give the organization a great deal of satisfaction.	0.875			
Subjective standards (NS)	NS1	Business decision-makers believe that the organization should recycle waste.	0.890	0.845	0.893	0.772
	NS2	People within the organization think that we should be involved in recycling.	0.959			
	NS3	People outside our organization think that our organization should engage in recycling.	0.971			
	NS4	Many organizations like our organization are involved in recycling.	0.825			
	NS5	Neighborhood organizations practice waste recycling	0.882			
Perceived behavioral control (PBC)	TCP1	The organization knows what can be recycled	0.859	0.952	0.937	0.73
	TCP2	The organization knows where to take the waste for recycling.	0.856			
	TCP3	Our organization knows how to recycle	0.966			
	TCP4	Whether the organization recycles is entirely up to us	0.944			
	TCP5	Whether the organization recycles effectively is entirely within our control.	0.894			
Intent (INT)	INT1	Our organization intends to recycle	0.892	0.822	0.885	0.755
	INT2	Our organization intends to Reduce waste generation	0.830			
	INT3	Our organization is willing to reuse the waste it generates	0.907			
	INT4	Our organization is willing to participate in the recycling chain.	0.976			
Circular economy capabilities	CEC1	Our organization uses environmentally friendly raw materials	0.827	0.928	0.971	0.763
	CEC2	Our organización separates waste	0.925			
	CEC3	Our organization delivers generated waste to a waste management company.	0.904			
	CEC4	Our company reuses the waste generated	0.865			
	CEC5	Our company works to reduce waste generation.	0.880			
Adoption of BD (ABD)	ABD1	Our company intends to adopt BD	0.870	0.959	0.819	0.784
	ABD2	Our company intends to start using the DB on a regular basis in the future.	0.819			
	ABD3	Our company would recommend the adoption of BigData to others.	0.967			
Relative advantage (ROA)	VR1	The BD enables our company to properly manage waste	0.984	0.971	0.974	0.714
	VR2	The BD enables our company to minimize all types of waste.	0.904			
	VR3	BD would enable our company to respond more quickly than competitors to changes in the environment.	0.916			
Compatibility (COMP)	COMP1	The use of BD is consistent with our business practices.	0.988	0.813	0.863	0.628
	COMP2	The use of the DB is in line with our organizational culture.	0.824			
	COMP3	In general, it is easy to incorporate the DB in our company.	0.938			
Security (SEG)	SEG1	The need to outsource DBs raises concerns about data security and privacy.	0.982	0.856	0.925	0.651
	SEG2	The need to outsource DBs creates vulnerability in controlling access to corporate information.	0.945			
	SEG3	The need to outsource the DB creates risks due to over-dependence on the supplier.	0.935			
Senior management support (AD)	AD1	Our top management promotes the use of DB in the company.	0.850	0.928	0.905	0.703
	AD2	Our senior management builds support for BD initiatives within the company.	0.820			
	AD3	Our top management promotes BD as a strategic activity within the company.	0.844			
Organizational readiness (OP)	PO1	Lack of capital/financial resources has prevented my company from fully exploiting the BD.	0.958	0.811	0.966	0.627
	PO2	The lack of analytical capacity prevents the company from fully exploiting the DB.	0.961			
	PO3	Lack of skilled resources prevents the company from fully exploiting the DB.	0.926			
Competitive Pressure	PC1	Adopting BD would be heavily influenced by what competitors in the industry are doing.	0.966	0.957	0.883	0.771
	PC2	Choice to adopt BD depends on what competitors do	0.841			
	PC3	I would adopt BD in response to what competitors are doing.	0.875			
Government support (GA)	AG1	Government policies encourage our company to adopt new technologies such as BD.	0.860	0.972	0.986	0.705
	AG2	The government provides incentives for the adoption of BD in public procurement and contracts.	0.852			
	AG3	Standards or laws support the adoption of BD technologies	0.937			

^1^Indicator loadings > 0.5 indicate the reliability of the indicator (Hulland, 1999, p. 198). ^2^Cronbach's alpha > 0.7 indicates internal consistency of a set of indicators (Nunnally, 1967). ^3^Composite reliability (CR) > 0.7 indicates the internal consistency of a set of indicators (Gefen et al., 2000). ^4^Average variances extracted (AVE) > 0.5 indicates convergent reliability (Fornell and Larcker, 1981; Bagozzi and Yi, 1988).

Regarding internal consistency, the literature indicates that Cronbach's alpha and composite reliability (CR) values must be greater than a minimum of 0.70 (Hair et al., 2011). In this study, it was found that both Cronbach's alpha and the CR values exceeded the minimum values recommended by the literature, ranging between 0.863 and 0.974, for Cronbach's alpha and 0.822 and 0.971 for CR (see Table 2). Consequently, it can be inferred that the study complies with the internal consistency criterion; similarly, the study met the convergent validity criterion in that the AVE was greater than 0.5 for each construct (see Table 2).

#### 5.1.1. Discriminant validity using SEM-PLS

To assess discriminant validity, the Fornell and Larcke (1981) was used, according to which the square root of the AVE of each construct must be greater than its correlation with other constructs (Chin, 2010). The results indicate that the measurement model met the Fornell-Larcker criterion (see Table 3) and finally, it was found that there was no multicollinearity problem, since the variance inflation factor (VIF) of the constructs and indicators in all cases were less than 3.0 (Hair et al., 2017). Compliance with the criteria established in the literature allows us to establish that the model is adequate, therefore, we proceed to evaluate the structural model.

**Table 3:** Discriminant validity (Fornell-Larcker criterion).

**Scale**	**Act**	**NS**	**TCP**	**INT**	**CEC**	**VR**	**COMP**	**SEG**	**AD**	**PO**	**PC**	**AG**
Act	**(0,784)**											
NS	0,625	**(0,885)**										
TCP	0,556	0,589	**0,815**									
INT	0,703	0,347	0,485	**0,874**								
CEC	0,697	0,423	0,415	0,360	**0,831**							
VR	0,714	0,554	0,221	0,348	0,388	**0,782**						
COMP	0,231	0,406	0,283	0,482	0,414	0,705	**0,782**					
SEG	0,210	0,527	0,411	0,562	0,591	0,636	0,433	**0,809**				
AD	0,413	0,686	0,561	0,302	0,569	0,329	0,647	0,434	**0,847**			
PO	0,338	0,243	0,249	0,564	0,376	0,225	0,574	0,296	0,557	**0,781**		
PC	0,677	0,304	0,699	0,559	0,300	0,333	0,578	0,633	0,339	0,490	**0,814**	
AG	0,313	0,283	0,609	0,320	0,278	0,529	0,645	0,333	0,535	0,524	0,625	**0,838**

The diagonal values (in bold) are the square root of the AVEs of the latent variables and indicate the highest in any column or row.

### 5.2. Structural model

For the structural model, the predictive power of the constructs R2 was examined. Cohen (1992), states that an R2 of 0.02, 0.13 and 0.26 can be considered as small, medium, and large respectively. The R2 values for behavioral intentions (INT), CE behavior's (CEC) and BD were 0.220, 0.245 and 0.204 respectively, which confirmed that the estimates fit the data well and have high predictive power (Table 4).

**Table 4:** Predictive relevance.

**Scale**	**R2**	**Q2**
INT	0.220	0.537
CEC	0.245	0.334
ABD	0.204	0.444

For predictive relevance, the Stone-Geisser's Q2 was used, a criterion to evaluate the cross-predictive relevance of the PLS trajectory model, which according to Hair et al. (2019), values greater than 0, 0.25 and 0.50 represent small, medium, and large predictive relevance respectively. In the literature, a value for the omission distance between 5 and 12 is recommended (Hair et al., 2017). In the case of this research and skip distance of seven (D=7) was used (Ringle et al., 2015). The Q2 of behavioral intention (INT), CE behavior (CEC) and DB adoption in the study were 0.537, 0.344 and 0.444 respectively, confirming the predictive relevance of the structural model. On the other hand Hair et al. (2017) suggest that to achieve model fit, the SRMR value should be < 0.08, according to the above, it was found that the SRMR value is equal to 0.041, therefore it can be affirmed that the model satisfies the general fit criteria of PLS-SEM model.

### 5.3. Bootstrapping

Finally, the bootstrapping technique was used to evaluate path coefficients (standardized beta), the significance levels and the *t*-values (Ringle et al., 2015). Bootstrapping is a non-parametric technique generally used to test whether path coefficients (beta) are significant (Streukens and Leroi-Werelds, 2016), in which case, the PLS path model is estimated, in this case 5,000 times. The results indicated that the values are significant (*p* < 0.01) and can be seen in Table 5.

**Table 5:** Hypothesis testing (bootstrapping).

**Relations**	**Std. Beta**	**Std. Error**	***t* Values**	***p* Values**	**Results**	**95% LL CI**	**95% UL CI**
Act –> INT	0,255	0,038	3,712^***^	0,001	Supported	0,203	0,458
NS –> INT	0,497	0,030	2,000^***^	0,000	Supported	0,191	0,688
TCP –> INT	0,444	0,042	2,982^***^	0,005	Supported	0,275	0,719
INT–>CEC	0,416	0,046	1,523^**^	0,005	Supported	0,036	0,452
ABD–> CEC	0,225	0,047	3,315^*^	0,003	Supported	0,135	0,360
VR–> ABD	0,311	0,044	1,468^*^	0,004	Supported	0,140	0,451
COMP–> ABD	0,219	0,035	2,135^*^	0,002	Supported	0,156	0,375
SEG-> ABD	0,299	0,036	2,649^*^	0,000	Supported	0,038	0,337
AD–> ABD	0,356	0,040	2,495^*^	0,003	Supported	0,003	0,359
PO–> ABD	0,305	0,046	3,856^*^	0,000	Supported	0,282	0,587
PC–> ABD	0,213	0,049	1,676^*^	0,000	Supported	0,024	0,237
AG–> ABD	0,434	0,050	3,532^*^	0,001	Supported	0,170	0,604

^*^p < 0.1; ^**^p < 0.05; ^***^p < 0.01; t-values around 1.65, 1.96 and 2.58 are considered at the 10%, 5% and 1% significance level, respectively (two-tailed test).

The direct effects of attitudes (Act), subjective norms (NS) and perceived behavioral control (TCP) on behavioral intentions (INT) respectively, turned out to have significant values of 0.255, 0.497 and 0.444 (*p* < 0.01). Therefore, hypotheses 1, 2 and 3 are empirically accepted (see Table 5). The direct effects of behavioral intentions (INT) and BD adoption (ABD) on EC behavior's (CEC) respectively have significant values of 0.416 (*p* < 0.05) and 0.225 (*p* < 0.01), therefore, hypotheses 4 and 12 are also empirically accepted (see Table 5). The direct effects of the TOE model on BD adoption have significant values between 0.206 and 0.434 (*p* < 0.1), for the hypotheses, so they are also empirically supported (see Table 5).

**Figure 2:**
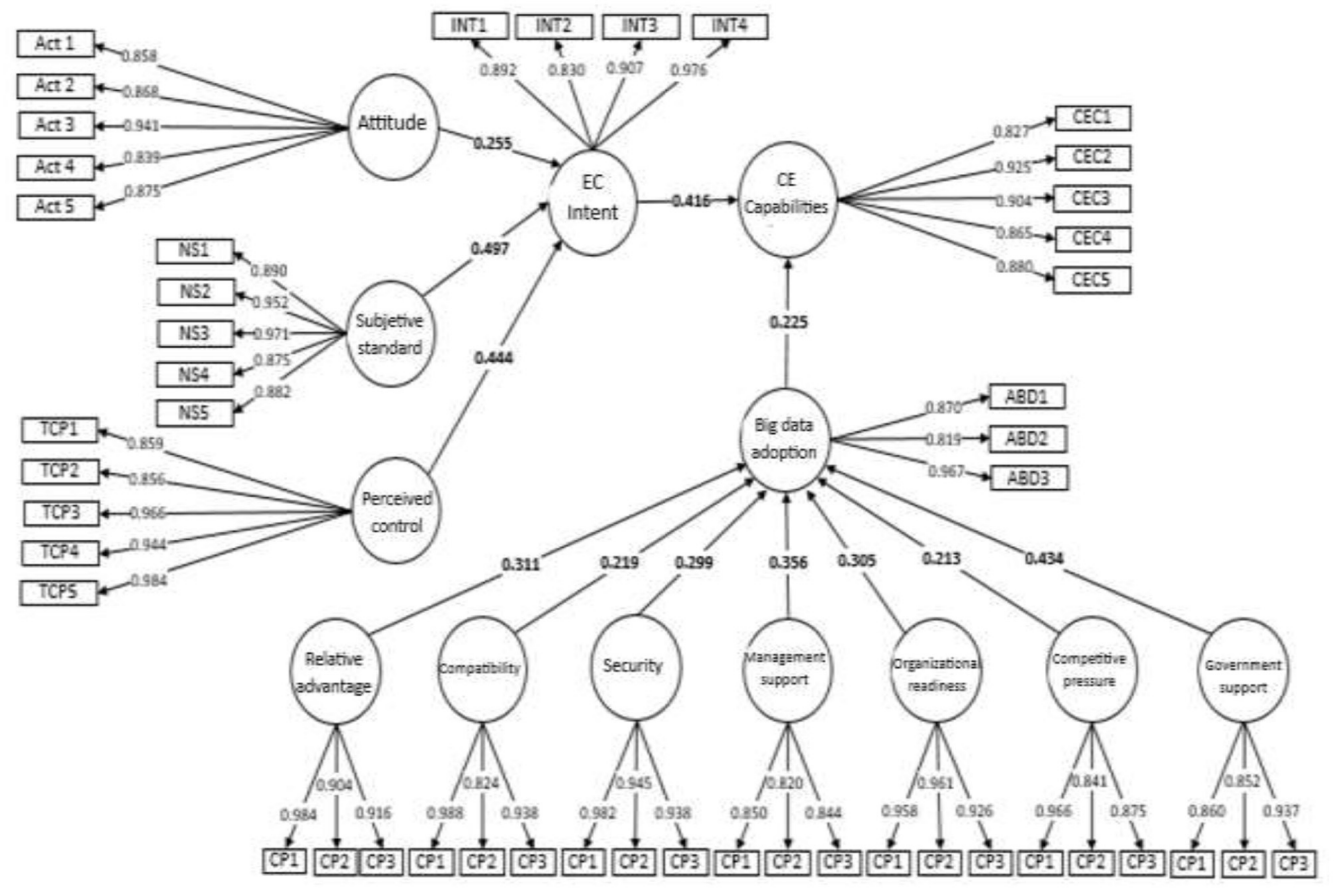
Research model tested.

Figure 2 presents the final research model tested. The results confirm that attitude, subjective norm and perceived behavioral control through intentions toward the circular economy influenced BD adoption. In addition, factors from the TOE model also significantly influenced BD adoption.

## 6. Discussion

The present study aimed to assess the influence of attitudes, subjective norms, and perceived behavioral norms on behavioral intentions toward CE and the adoption of BD by companies in Colombia. To this end, we sought to ensure the discriminant validity and reliability (internal consistency: Cronbach's alpha coefficient and composite reliability) of the instrument, which exceeded the minimum values allowed according to the literature, showing that the questionnaire was valid, reliable and statistically relevant, in addition, the model explained the variables that describe behavior with respect to CE and BD adoption.

Intention and behavior toward CE in BD environments were the gap that the study aimed to close. The study shows that attitude is an important determinant of behavioral intention, which is consistent with previous studies (Chuang et al., 2018; Moghimehfar et al., 2018; Sharma and Foropon, 2019; Elzinga et al., 2020; Lin and Roberts, 2020; Yang et al., 2020). The model can confirm that perceived behavioral control has the most significant impact on CE intention, corroborating previous studies in which both individual and organizational factors affect these pro-environmental behavior's (Yuriev et al., 2020). Another important aspect is that the model explains 64.3% of the dependent variable.

The results clearly identify that attitudes, subjective norms and perceived behavioral control are strong determinants of organizations' intentions to implement recycling best practices, with attitudes being the strongest predictor of intentions. Furthermore, these intentions also largely determine organizations' behavior's toward circular economy competence.

The results confirm the findings of Ajzen (1991), Cordano and Frieze (2000), Papagiannakis and Lioukas (2012), Botetzagias et al. (2015); Thoradeniya et al. (2015); Singh et al. (2018); Khan et al. (2020a) indicating that attitude is an important determinant of behavioral intention and carries greater weight than subjective norm and perceived behavioral control.

The study also identified that more than 80% of the participants agree that recycling is the responsibility of their organizations, but only 45% perceive that recycling would bring great satisfaction to their organizations. In addition, 32% of respondents reported that they do not know how their organizations can contribute to recycling which may limit the adoption of CE; to overcome this difficulty, the government could not only generate more benefits in terms of CE behavior, but also generate more awareness and compliance campaigns on waste management especially in SMEs (Botetzagias et al., 2015).

In addition, the study revealed that 83% of the respondents showed positive aggregate perceived behavioral control. However, 22% of the respondents noted that they do not know how their organizations can contribute to recycling. This finding opens the possibility for the government to increase efforts to further disseminate knowledge regarding recycling, particularly in small organizations, to achieve a CE for waste (Khan et al., 2020a).

One element worth highlighting is that while the perception of the involvement of neighboring companies in CE activities is relatively low (26%), 88% of the participants perceive that the majority of people within their organizations are in favor of participating in CE activities.

The above results, while corroborating the importance of TCP related to pro-environmental behavior (Singh et al., 2018; Khan et al., 2020b), cannot be generalized as they are based on the culture of a respective country (Heidbreder et al., 2019).

About the statement “Recycling is fun”, the results identify the importance for managers on the grounds that circularity can be seen to go beyond the simple evaluation of good results or even feelings of usefulness. Recognizing its value in terms of generating profit will maintain interest in process modification, as it assumes that the investment can ultimately be recouped through process optimization (Khan et al., 2020a; Warmington-Lundström and Laurenti, 2020).

Satisfaction captured with the statement “Recycling waste from will bring great satisfaction to our organization” shows favorability with CE as implementation saves time and materials, streamlines processes and allows for more competitive products to be created (Singh and Singh, 2018; Clube and Tennant, 2020).

The phrase “Our organization is responsible for recycling waste” has a significant impact on the intention to accept CE, as managers recognize that by producing products for the market, they are also responsible for dealing with waste beyond the regulatory requirements (Friedrich, 2020; Mitrano and Wohlleben, 2020; De Tandt et al., 2021).

Companies recognize that various external and internal stakeholders expect companies to take care of their waste, which obliges companies to implement CE (Reijonen et al., 2021).

Recognition of the goodness of recycling in turn has a positive impact on customer acceptance of the company and enhances reputation, which can be reflected in sales preferences and change consumer behavior. However, when asked about the activities of other organizations related to CE, the vast majority responded that these organizations are not very involved. Perhaps this involvement exists but seems less evident.

### 6.1. Conclusions

The main contribution of this study was to improve the understanding of the factors influencing organizations' intentions and behavior toward CE in BD environments. The analytical approach used in this article was a strength as it allows the estimation of the correlation between variables through multivariate analysis using structural equation modeling with least squares equations (SEM PLS).

It was found that the variables of norm, subjective, attitude and perceived control have significant influence on CE intention and with this intention it is possible to precede behavior's associated with CE capabilities. In this regard, it was identified that although most participants have positive intentions toward CE, most organizations do not seem to do so. This may be due to a lack of incentives, limited knowledge on how to engage favorably in the CE process or the non-existence of networks for inter-company collaboration. To overcome this intention-behavior gap and move toward CE, the government should generate greater incentives, socialize and train on circular economy issues and create platforms or networks for collaboration between companies.

There is no doubt that there is a clearly positive will and intention toward a more restorative and sustainable corporate strategy. However, there are significant problems and limitations in the application of the CE model. In this respect BD has evolved into a tool to resolve these intricate operational issues and develop fundamental models for sound CE decision making.

Designing CE adoption strategies is highly dependent on the volume and veracity of BD (Lopes de Sousa et al., 2018). In this regard, it is vital to examine the organization's current capacity in relation to BD, as this suggests the organization's potential operational capacity in the future. One element to contemplate in this scenario is that BD adoption remains difficult, largely due to the ambiguity and uncertainty surrounding future business functionality (Despeisse et al., 2017; Murray et al., 2017). However, the knowledge derived from DB analytics is perceived as an enabler in terms of reducing uncertainties and generating prediction-based outcomes, which facilitates decision making for organizations with respect to CE. The knowledge gained from analytics will enable the organization to simplify complex processes and thus improve the long-term sustainability of operations (Murray et al., 2017). In the case of the CE paradigm, DB functionalities can be used to generate information to integrate processes and share resources. It can also be used to analyze consumption patterns and supply-side variability, which would allow redesigning processes for a cyclical rather than linear approach.

This study should be replicated in different nations and different sectors and types of companies to better understand the implementation of the circular economy and which variables have the greatest influence on it.

#### 6.1.1. Theoretical implications

Being a relatively new idea in the literature, the factors that define CE behavior are currently being tested. Due to the scarcity of studies addressing the issue in Latin American firms, and especially in Colombia, the contribution of this study is relevant given that some of this literature is linked to manufacturing processes and even consumer expectations. The use of an approach to evaluate firms based on the notion of planned behavior is novel, however, it is recognized that given factors such as technology or legislation vary by country, it is predicted that firms' results may vary slightly. Results measuring both the correlation between variables and re-editing to explain behavior with respect to the circular economy have been obtained using SEM-PLS.

#### 6.1.2. Practical implications

The CE approach is a relatively new concept that offers a restorative and economically viable approach to doing business with triple benefits: resource conservation, environmental protection, and economic benefits (Lieder and Rashid, 2016). Adopting a CE involves considerable changes in organizations' operations, which can foster innovation by finding newer and more efficient alternatives for redesigning, reusing or upgrading products, recycling available resources or managing waste (Abbate et al., 2022). Studies have suggested that a sustainability-based approach is facilitated by the development of a shared vision and a collaborative stance among all stakeholders along with a clear explanation of long-term responsibilities (Geissdoerfer et al., 2017).

With the emergence of COVID-19, safety protocols in waste management became even more relevant as a determining factor in containing the spread, consistent with the principles and approach of the circular economy, and it is recognized that more and more companies could become interested in the implementation of the circular economy.

For managers, the CE and BG approach suggests rethinking even their basic assumptions regarding the use and development of new products, the integration of their supply chains, reverse logistics, the scope of their responsibilities and above all the redesign of their business models in a way that can increase their organizational and environmental efficiency. These elements of course align with meeting the SDGs, which are an ongoing concern for the world's sustainability. For governments this has even more implications, as it implies in addition to the above challenges a coordinated agenda between public administration, economic sectors and society to encourage the transition from a linear economy to a circular economy by promoting elements such as sustainable sourcing, eco-design, industrial symbiosis, economy of functionality, responsible consumption, product life cycle extension and efficient end-of-life management of products and materials.

## 7. Limitations and future research directions

This study was conducted in a developing or emerging economy in South America (Colombia), where technology adoption in SMEs is at an embryonic stage, because the scenario may be different in other countries even in the same region, so future researchers should interpret their findings in the light of the conditions of each context.

In this sense the study, too, has some limitations, related to specific cultural and social contexts, which makes the results not generalizable (Thoradeniya et al., 2015). Furthermore, although recommendations to ensure data quality such as anonymity and self-administration of the questionnaire were followed, social desirability or politically correct response bias cannot be ruled out, in the sense that respondents' perceptions may not coincide with objective and rational reality. Another element that limits the study is that it was done cross-sectionally, a longitudinal study could show whether the elements found are sustained over time. nevertheless, the study provides an opportunity for future research, e.g., replication in other emerging economies for comparative studies. technology, size or sector as mediators of circular economy behavior could also be addressed.

Future lines of research could examine business models for the transition to the collaborative digital economy and deepen the understanding of how companies can attract and add value to their customers. Other studies could characterize the dynamic capabilities of firms and the application of CE at local, regional, national and international levels, so that comparisons can be made with other countries and sectors. Finally, there is a need to promote studies on sustainable sourcing, eco-design, industrial symbiosis, economy of functionality, responsible consumption, product life cycle extension and efficient end-of-life management of products and materials.

## Data availability statement

The original contributions presented in the study are included in the article/supplementary material, further inquiries can be directed to the corresponding author.

## Ethics statement

Ethical approval was not required for the study involving human participants in accordance with the local legislation and institutional requirements. Written informed consent to participate in this study was not required from the participants in accordance with the national legislation and the institutional requirements.

## Author contributions

Conceptualization: CA and MP. Methodology and writing—preparing the original draft: CA, MP, MGa, and MGo. Validation: CA. Data curation: CA and MGo. Writing—revising and editing: AM. All authors have read and accepted the published version of the manuscript.
